# Fighting the War Against COVID-19 via Cell-Based Regenerative Medicine: Lessons Learned from 1918 Spanish Flu and Other Previous Pandemics

**DOI:** 10.1007/s12015-020-10026-5

**Published:** 2020-08-13

**Authors:** You Jeong Park, Jeffrey Farooq, Justin Cho, Nadia Sadanandan, Blaise Cozene, Bella Gonzales-Portillo, Madeline Saft, Maximillian C. Borlongan, Mia C. Borlongan, R. Douglas Shytle, Alison E. Willing, Svitlana Garbuzova-Davis, Paul R. Sanberg, Cesar V. Borlongan

**Affiliations:** 1 Department of Neurosurgery and Brain Repair, University of South Florida Morsani College of Medicine, 12901 Bruce B Downs Blvd, Tampa, FL 33612 USA; 2 Georgetown University, 3700 O St NW, 20057 Washington, DC USA; 3 Tulane University, 6823 St. Charles Ave, 70118 New Orleans, LA USA; 4 Northwestern University, 633 Clark St, 60208 Evanston, IL USA; 5 University of Michigan, 500 S State St, 48109 Ann Arbor, MI USA; 6 Tampa Preparatory School, 727 West Cass St, 33606 Tampa, FL USA; 7 University of California Berkeley, 94720 Berkeley, CA USA

**Keywords:** COVID-19, Coronavirus, Pandemics, SARS Virus, Vaccines, Antiviral Drugs, 1918 Influenza Pandemic, Stem Cells

## Abstract

The human population is in the midst of battling a rapidly-spreading virus— Severe Acute Respiratory Syndrome Coronavirus 2, responsible for Coronavirus disease 2019 or COVID-19. Despite the resurgences in positive cases after reopening businesses in May, the country is seeing a shift in mindset surrounding the pandemic as people have been eagerly trickling out from federally-mandated quarantine into restaurants, bars, and gyms across America. History can teach us about the past, and today’s pandemic is no exception. Without a vaccine available, three lessons from the 1918 Spanish flu pandemic may arm us in our fight against COVID-19. First, those who survived the first wave developed immunity to the second wave, highlighting the potential of passive immunity-based treatments like convalescent plasma and cell-based therapy. Second, the long-term consequences of COVID-19 are unknown. Slow-progressive cases of the Spanish flu have been linked to bacterial pneumonia and neurological disorders later in life, emphasizing the need to reduce COVID-19 transmission. Third, the Spanish flu killed approximately 17 to 50 million people, and the lack of human response, overcrowding, and poor hygiene were key in promoting the spread and high mortality. Human behavior is the most important strategy for preventing the virus spread and we must adhere to proper precautions. This review will cover our current understanding of the pathology and treatment for COVID-19 and highlight similarities between past pandemics. By revisiting history, we hope to emphasize the importance of human behavior and innovative therapies as we wait for the development of a vaccine.

**Figure:**
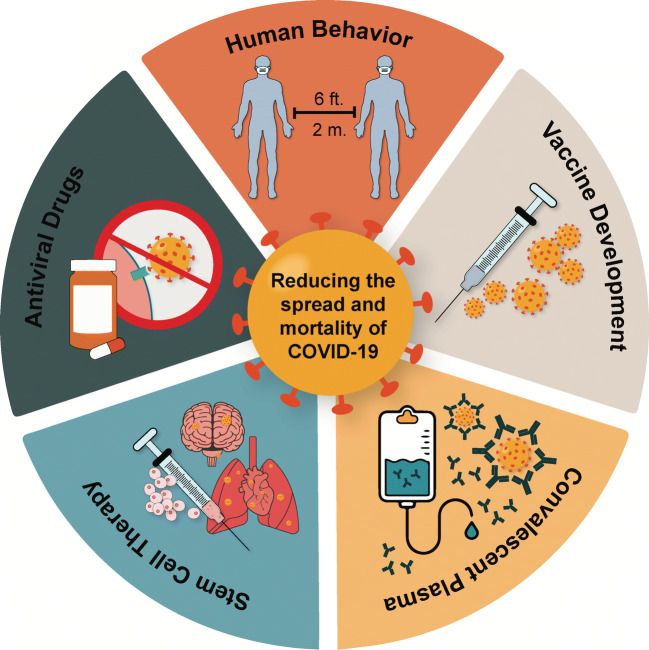
Graphical Abstract

Graphical Abstract

## Hindsight is 20/20

Global shutdowns, widespread reduction in economic activity, and stalls in the workforce and education systems are just a few of the major historic events that have amalgamated in 2020 due to the outbreak of the COVID-19 pandemic. According to the data collected by Johns Hopkins University, there are more than 8 million confirmed coronavirus cases and 437,500 deaths worldwide as of June 15, 2020 [1]. The US currently leads the world with more than 2.1 million confirmed cases and 116,130 deaths—steadily reaching Dr. Anthony Fauci of the National Institute of Allergy and Infectious Disease projected number of 100,000 to 240,000 nation-wide deaths back in March of 2020.

History repeats itself and when assessing the proper responses to the current pandemic, it is important to learn from the past. In a historical review of viral pandemics, the 1918 Spanish flu stands out which infected 500 million people and led to an estimated 17 to 50 million deaths [2]. Like the current pandemic, the Spanish flu began from a zoonotic transmission, progressed rapidly from infection to symptom onset with viral consolidation in the lung, and lead to death primarily by pneumonia in severe cases [3, 4]. The main catalyst of the high mortality rate of the Spanish flu was lack of initial response, overcrowding, and poor hygiene, emphasizing the critical role human behavior plays in the spread of infectious diseases. Three important takeaways stand out from the 1918 pandemic. First, those who survived the first wave developed immunity against the virus and gave these individuals immunity during the second wave. This highlights the potential of passive immunity-based treatments using antibodies collected from the plasma of recovered COVID-19 patients. The second lesson is that the end of Spanish flu cases in 1920 marking the eradication of the pandemic may not capture the full picture of the pandemic. Evidence of slow-progressive cases of Spanish flu has been associated with secondary bacterial pneumonia linked to neurological disorders like pediatric encephalitis lethargica in the 1920 s [5], which was suggested to manifest in adulthood as Parkinson’s disease [6]. Similarly, the potential long-term neurological and cardiac repercussions of COVID-19 warrant critical attention as the research surrounding this pandemic unfolds. Third, the overcrowding in military camps and lack of sanitation due to poverty during World War I contributed to the escalation of the Spanish flu, emphasizing the importance of human behavior on containing the current pandemic. As states reopen in response to the current economic crisis and restrictions continue to ease over the next few months, the security blanket of disillusioned comfort—or ignorance of the dangers of the virus—may be the biggest threat to the future health of our society.

This review will cover our current understanding of the pathology of COVID-19 and highlight similarities between the Spanish flu and past pandemics’ pathology, social behavior, and treatments. By focusing on these disease overlaps, in particular convalescent plasma and stem cells targeting the virus entry point, we may gain insights on the best treatments and human behavior to address the current pandemic. Furthermore, we will discuss the latest preclinical and clinical data of available therapies and by highlighting critical gaps in knowledge, we hope to emphasize the importance of human behavior and innovative therapies as we wait for the development of a vaccine.

## The War Against COVID-19

### Binding of Spike Protein and Human Angiotensin-converting Enzyme 2: the Shot Heard Around the World

The term coronavirus refers to more than 40 species of viruses within the family *Coronaviridae* that cause respiratory tract infections in humans. Coronaviruses classify into either alpha and beta subtypes that infect humans, or into gamma and delta subtypes, which mainly infect avians. Severe Acute Respiratory Syndrome Coronavirus (SARS-Cov) and Middle East Respiratory Syndrome-related Coronavirus (MERS-Cov), are two betacoronaviruses that precipitated the SARS and MERS epidemics in 2003 and 2012, respectively. Severe Acute Respiratory Syndrome Coronavirus 2 (SARS-Cov-2) is a new genetically-related betacoronavirus species responsible for the COVID-19 pandemic.

**Fig. 1:**
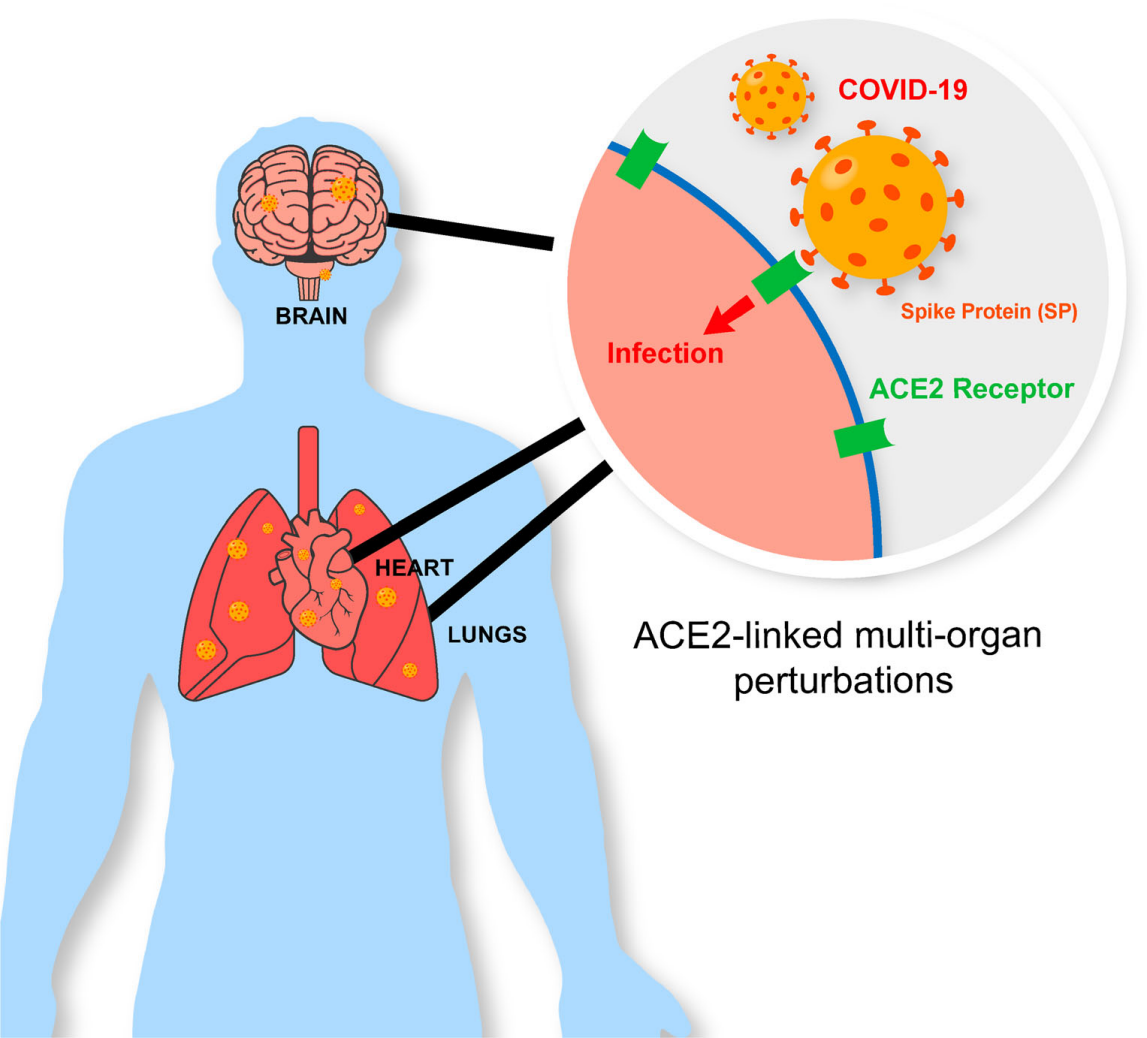
The trimeric spike protein of SARS-Cov-2 mediates the entry of the virus into host cells via the ACE2 receptor. The ACE2 receptor is present in various organs throughout the body making multi-organ infection possible

SARS-Cov-2 is a single-stranded, non-segmented, positive-sense RNA virus that measures 65–125 nm in diameter and 29.9 kb in length [7, 8]. It is composed of a 5’-untranslated region (UTR), 3’-UTR, replicase complex, membrane protein gene, nucleocapsid gene, envelope protein gene, and spike protein gene [9]. The trimeric spike protein (SP) of SARS-Cov-2 mediates the entry of the virus into host cells and is therefore critical to the pathogenesis of the disease (Fig. 1). SP is a class one dual subunit transmembrane fusion glycoprotein exclusively expressed by viruses that binds to human angiotensin-converting enzyme 2 (ACE2), an endogenous extracellular receptor. This initiates cleavage events that facilitate SARS-Cov-2 fusion and entry into the host cells [10]. The S1 and S2 subunits of SP are non-covalently bound, host-processed proteins that mediate the binding and fusion capabilities of SARS-Cov-2, respectively [10–12]. The S1 subunit recognizes and binds to ACE2 with high affinity, implicating the interaction of viral SP and human ACE2 as the key intermediary for host-cell entry [13].

ACE2 is a type 1 integral membrane protein that functions as an extracellular receptor attached to the plasma membrane of cells in the lungs, heart, brain, and other vital organs [14, 15]. It is primarily expressed on type 2 pneumocytes in the lungs and on enterocytes in the small intestine. ACE2 normally modulates blood pressure through the renin-angiotensin system by cleaving circulating angiotensin II into the vasodilator angiotensin. SARS-Cov-2 leverages the ubiquitous expression and physiologic importance of ACE2 to enter and replicate within healthy cells throughout the body. This leads to an increase in viral load and systemic adverse multi-organ effects.

The potent interaction between ACE2 and SP mediates the efficient entry of the virus into host cells. Electrostatic interactions between the negatively charged ridges that flank the catalytic domain of ACE2 and the positively charged receptor-binding domain (RBD) of SP enable SARS-Cov-2 to localize to its receptor [16]. ACE2 receptors contain a peptidase domain that binds to the RBD of SPs through polar interactions [17]. Following this docking, two proteolytic cleavage events take place. First, furin protease cleaves SP into S1 and S2 subunits. Second, the serine protease TMPRSS2 cleaves SP on the S2 subunit, initiating a series of conformational changes that prime the virus to enter the cell [18, 19]. TMPRSS2 also cleaves ACE2 between amino acids 697 to 716, and this cleavage is crucial to enhancing viral uptake [20]. The metalloprotease ADAM17 also acts on ACE2, but this does not augment viral uptake [20]. Therefore, TMPRSS2 both activates SARS-Cov-2 for uptake and simultaneously amplifies the rate of its uptake by cleaving ACE2.

After binding and proteolytic processing, the final step for infection is the viral entry into the host cell. It is not entirely clear how SARS-Cov-2 enters the cell, as there is evidence for both fusion and endocytosis. In a fusion-based entry model, SARS-Cov-2 fuses its envelope with the plasma membrane of the host cell and empties its genetic contents into the cytosol, where it hijacks the host machinery to replicate its genes and produce more virus. In the endocytosis model, SP interacts with ACE2 to cause a pH-dependent internalization of the virus-receptor complex [21]. On the other hand, the endocytic-uptake of SARS-Cov-2 is independent of clathrin and caveolae-based pathways [21]. Clathrin-mediated endocytosis involves the formation of vesicles coated by the protein clathrin that form following internal budding of the plasma membrane. Caveolar endocytosis occurs when oligomerized caveolin membrane form scaffolds that invaginate into endocytic vesicles. The precise type of endocytosis that results in the internalization of SARS-Cov-2 is unknown but does not involve these two mechanisms.

The SP gene of SARS-Cov-2 determines its affinity for ACE2, in contrast to the SPs of related coronaviruses with slightly different sequences that favor binding to other targets. Each cycle of viral replication within the host cell produces additional copies of the viral RNA genome, including the regions that encode this specific SP variant. Reverse-transcriptase polymerase chain reaction (RT-PCR) recognizes the SP of SARS-Cov-2 and measures total viral load. This technique utilizes a primer that binds to regions of the SARS-Cov-2 RNA genome, including the SP region, and amplifies the amount of virus present such that the fewer cycles it takes to reach a threshold value indicates a higher initial concentration of SARS-Cov-2. SP expression and viral load are highest at the onset of infection and lowest during recovery [22]. Elevated viral load thus indicates increased infectivity due to the additional SP binding ACE2.

SP is integral for SARS-Cov-2 to recognize and internalize into cells. Viral binding and fusion with host cells are obliterated in the absence of SP expression. After uptake, the virus uses host cell ribosomes to replicate; however, the cell will eventually undergo apoptosis or immune system-mediated destruction [23]. Therefore, SARS-Cov-2 must continuously infect new cells using SP-mediated uptake mechanisms. SP is also crucial for SARS-Cov-2 to infect other tissues beyond the original site in the respiratory tract. The virus travels to other organs through the bloodstream and, upon SP binding, fuses with ACE2-expressing organs to establish secondary sites of infection. Thus, SP and ACE2 must co-exist for the virus to propagate its growth and remain contagious.

The critical interaction of SP and ACE2 as the gatekeeper of SARS-Cov-2 entry into host cells can be confirmed and exploited using human recombinant soluble ACE2 (hrsACE2). ACE2 is a transmembrane protein and is not present endogenously in a soluble form; therefore, SP always binds to cell-surface ACE2. However, the administration of hrsACE2 introduces soluble ACE2 into the circulation that can complex with free SARS-Cov-2, thus blocking its ability to bind to cell-associated ACE2 [24]. The virus still binds to these functionally inactive receptors in a dose-dependent manner but does not undergo proteolytic activation or entry into the cell [24]. Importantly, SP binds to hrsACE2, but not to mouse recombinant soluble ACE2 [24]. To this end, the hrsACE2 proves the selective binding of SP to ACE2 and may be therapeutically valuable as an option to minimize the potential uptake of the virus.

The specific structural features and amino acid sequences of SP and ACE2 underlie the selectiveness of their binding and explain the preferential binding of SARS-Cov-2 to ACE2 and rbsACE2. SP makes direct contact with ACE2 at three different points. In the first region of interaction, Gln^498^, Thr^500^, and Asn^501^ on the RBD interact with Tyr^41^, Gln^42^, Lys^353^, and Arg^357^ on ACE2 [17]. In the second region, Lys^417^ and Tyr^453^ on the RBD associate with Asp^30^ and His^34^ on ACE2 [17]. The final point of contact is between Gln^474^ and Phe^486^ on the RBD and Gln^24^ and Met^82^ on ACE2 [17]. Substitutions of these amino acids may disrupt the affinity of the binding between SP and ACE2. Indeed, certain variants of the ACE2 allele exhibit weakened intermolecular interactions, pointing to the possibility of COVID-19-resistance [25]. Similarly, the level of expression of ACE2 may be an indicator of resistance to SARS-Cov-2 infection or a prognostic marker whereby increased expression correlates with higher infectivity. Leveraging this to identify high-risk populations could help slow the spread of the virus. In contrast, alterations in the amino acid sequence may also increase the ability of the virus to enter the cell by further stabilizing the SP-ACE2 interaction [26]. In addition to the RBD, amino acids 450–650 of SP are arranged in two anti-parallel β-sheets, β5 and β6. These SP structures, especially β6, bind with high affinity to ACE2, further emphasizing SPs’ importance as the ligand for ACE2 [27].

Although there are animal reservoirs of the virus, SARS-Cov-2 is most strongly associated with pathological effects in humans. Transgenic animal models expressing human ACE2 recapitulate its specificity and affinity for SP. Upon inoculation with SARS-Cov-2, mice expressing human ACE2 rapidly display signs of lethal infection in the lungs and brain, whereas mice expressing murine ACE2 do not develop infection [28]. In the transgenic mice, there is a rapid accumulation of immune cells, particularly macrophages, in the epithelia of the lungs and brain where they release proinflammatory cytokines and initiate a full immune response [28].

The capacity of SP to utilize human ACE2 to enter cells ensures the viability of the virus in several tissues. ACE2 expression is high in the small intestines, kidneys, heart, and lungs, although it is also present in key organs such as the nerves and brain [29]. RT-PCR analysis demonstrates the highest viral load in the lungs and intestines, with 3.6 × 10^5^ and 2.7 × 10^5^ copies per gram of tissue, respectively [30–32]. The prevalence of SP-ACE2 binding in these organs explains their substantial capacity to harbor the virus. It also justifies the elevated viral loads in those other organs such as the heart and brain.

The highly specific interaction between SP and ACE2 is well defined and is a critical target for therapeutic strategies that attempt to prevent infection. A variety of vital organs express the ACE2 receptor and are thus susceptible to SP-binding and the lethal damage induced by viral infection. Therefore, it is crucial to evaluate the effects of SARS-Cov-2 in the most commonly affected organ, the lungs, but also in other ACE2- and SP-expressing organs such as the brain, heart, intestines, and liver.

### Breathe Free or Get Sick Trying

The lungs are the primary site of infection and nearly all patients with COVID-19 present with some form of respiratory impairment. The most common complaints upon hospitalization are cough and shortness of breath, with 69% and 66% of patients reporting these symptoms, respectively. SARS-Cov-2 spreads through person-to-person contact or via small droplets, measuring less than 10 µm in diameter, that enter the respiratory tract after inhalation [33, 34]. As the virus traverses the airways, it binds to apical (luminal) ACE2 receptors via SP, then enters cells where it initiates replication. It preferentially infects cells of the alveoli, although it can also infect cells of the upper respiratory tract. Viral infiltration of the lung parenchyma directly precipitates the onset of severe acute respiratory syndrome (SARS). Despite the crucial role the respiratory tract plays in the transmission of SARS-Cov-2, airborne transmission is not an appreciable route of spread.

The lungs are the primary organ of the respiratory system and oversee numerous critical functions for survival and homeostasis, including acid-base balance and thermoregulation. They perform their primary role of gas exchange by drawing air into alveoli lined with pneumocytes. Oxygen diffuses across type 1 pneumocytes, which comprise 95% of the surface area of the alveoli. Type 2 pneumocytes are the most common cells in the alveoli, although they are small and do not constitute a large proportion of the total surface area. They secrete the pulmonary surfactant that prevents the alveoli from collapsing, and they can divide into type 1 or type 2 pneumocytes.

**Fig. 2:**
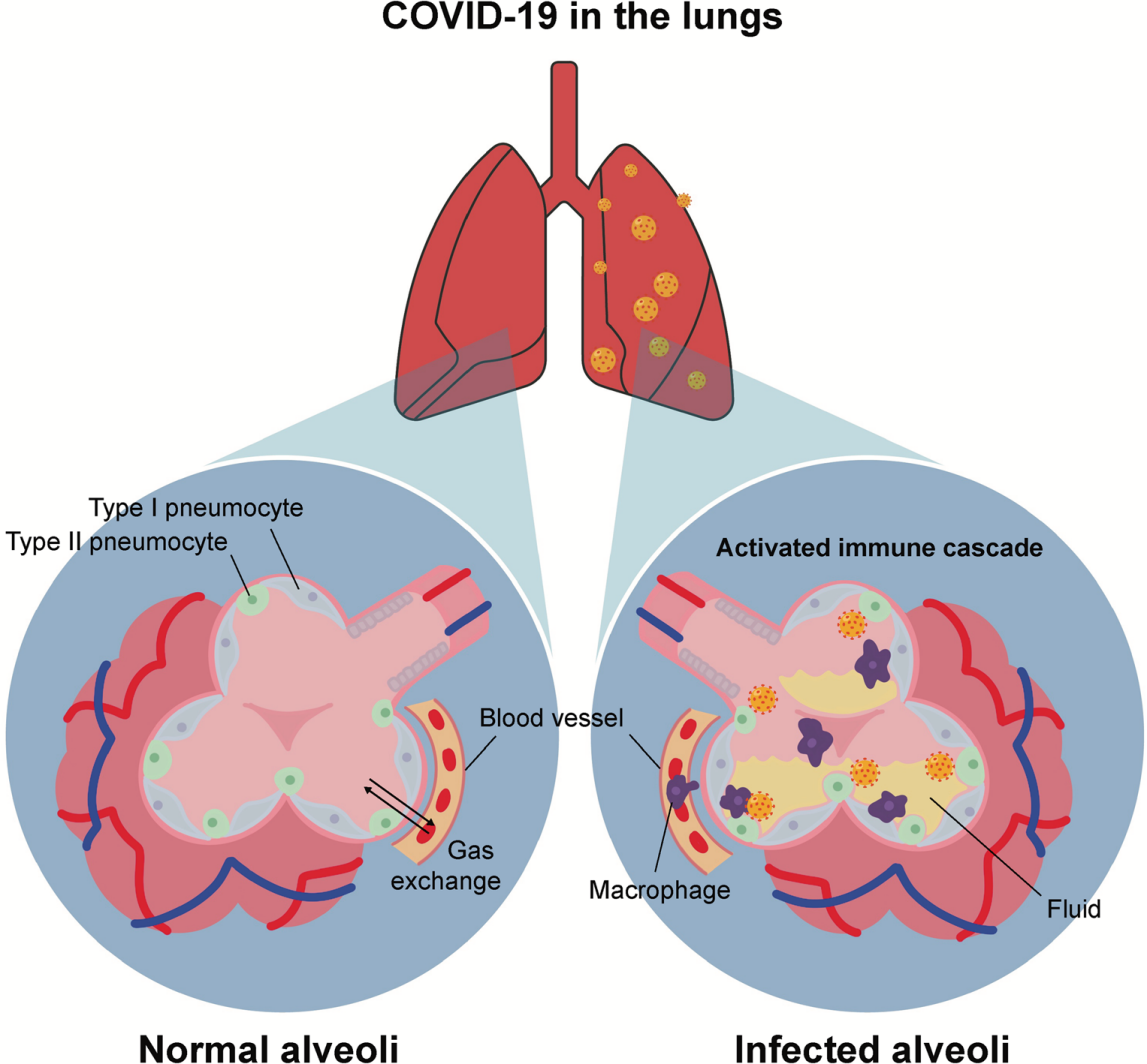
The coexpression of ACE2 receptors and TMPRSS2 transmembrane proteases by type 2 pneumocytes explains the susceptibility of the lungs to SP-mediated SARS-Cov-2 infection. Infection activates the immune cascade resulting in extensive tracheobronchial inflammation, diffuse alveolar damage, alveolar edema, pneumocyte desquamation, fibrin deposition, pneumocyte hyperplasia, and recruitment of alveolar macrophages

The coexpression of ACE2 receptors and TMPRSS2 transmembrane proteases by type 2 pneumocytes explains the susceptibility of the lungs to SP-mediated SARS-Cov-2 infection [35]. Well-differentiated type 2 pneumocytes express ACE2 more abundantly than poorly differentiated cells [36]. The targeting of these critical lung cells by SP has important functional consequences that manifest as SARS. SARS initially presents as fever, lethargy, pain, and cough, although it rapidly advances to shortness of breath and pneumonia. Macrophages of the innate immune system in the lungs likely detect SARS-Cov-A using RNA viral genome-recognizing RIG-like receptors, a specific type of toll-like receptor (TLR) present on dendritic cells and macrophages designed to complex with foreign molecules. Binding of the viral RNA to RIG-like receptors induces the recruitment of MyD88 and MAVS [37]. These two proteins promote NF-kB and type 1 interferon production culminating in the release of IL-6 and TNF alpha cytokines [37]. This immune cascade results in extensive tracheobronchial inflammation, diffuse alveolar damage, alveolar edema, pneumocyte desquamation, fibrin deposition, pneumocyte hyperplasia, and recruitment of alveolar macrophages **(**Fig. 2**)** [38]. Furthermore, a less severe form of pneumoconiosis known as anthracosis develops due to carbon accumulation [38]. SP interacts with ACE2 receptors on alveolar macrophages, both types of pneumocytes in the lungs, and the bronchial and submucosal gland epithelium of the upper airways [38]. After replication, SARS-Cov-2 preferentially exits cells via the apical surface, returning it to the lumen where it can continue to propagate through lung tissues [36].

The pathogenesis of SARS-Cov-2 produces severe respiratory outcomes within a few weeks of initial infection, even in the absence of preexisting respiratory comorbidities such as chronic obstructive pulmonary disease (COPD) [39]. In half of the critically ill patients, the development of SARS-Cov-2-induced pneumonia currently results in death within 28 days of diagnosis and 7 days of admission to the intensive care unit (ICU) [40]. Patients that require ventilators or who develop acute respiratory distress syndrome (ARDS), characterized by fluid accumulation in the alveoli, are more likely to die [40]. Approximately 67% and 71% of critically ill patients develop ARDS or require ventilator therapy, respectively [40].

One of the highest concentrations of ACE2 in the body is in the lung tissue. The potent binding of SP to ACE2 and subsequent cellular infection may explain the prevalence of respiratory symptoms in COVID-19 patients. Transgenic mice expressing hACE2 in the lungs exhibit a very similar pathology in the lungs compared to human patients, including pneumonia and macrophage infiltration; however, these respiratory symptoms are absent in wild-type mice [41]. Thus, the propensity of SARS-COV-2 to elicit lung-tissue damage and induce respiratory symptoms relies upon successful uptake into type 2 pneumocytes.

SARS-Cov-2 produces more severe symptoms in elderly patients than young patients, implicating an age-based loss of protection. Approximately 90% of infected pediatric patients are asymptomatic or exhibit mild symptoms [42]. Given the critical role of the lungs as the primary site of infection, this suggests there exist underlying physiological differences between adult and adolescent lung tissue that either predisposes adults to infection or protect adolescents.

While the severity of respiratory symptoms is useful in predicting outcomes, several non-invasive methods also allow tracking of the extent of COVID-19-induced lung damage. Computer Tomography (CT) analysis is a diagnostic imaging procedure useful for evaluating the pathology of the lungs. CT image-calculated well-aerated lung volume predicts the risk of ICU admission and death [43]. Thus, a more widespread SARS-Cov-2 lung infection correlates with worse outcomes. Ultrasound is another valuable imaging technique to explore the extent of lung damage. COVID-19 pneumonia increases the presence of B-lines, consolidation, and pleural line abnormalities in the posterior regions of the lungs [44]. Combined with its safety and efficiency, ultrasound imaging is valuable as both a diagnostic tool and as a follow-up measure to track changes in the extent of COVID-19 pneumonia in the lungs.

The interaction of SARS-Cov-2 with the lungs directly explains its tremendous infectivity potential. Not only is the respiratory tract is the primary point of entry of the virus into the body, but it also contains the highest concentration of ACE2 receptors for SP to complex with. Furthermore, SARS-Cov-2 survives within the respiratory tract more effectively than it can in other tissues, highlighting the imminent need for therapeutics targeting the lungs [45]. SP mediates this enhanced survival by downregulating ACE2 in the lungs [46]. Specifically, SP infects bronchoalveolar stem cells (BASCs) and controls BASC growth, inflammation, and ACE2 production [47]. SP downregulates ACE2 to self-limit its own infectivity. Excess virus production results in catastrophic lung damage and effectively destroys the tissues within which SARS-Cov-2 lives. SP-induced downregulation of ACE2 expression thus enhances viral survival, which increases the probability that SARS-Cov-2 will successfully transmit to another host. Simultaneously, ACE2 is also upregulated in the presence of lung tissue damage due to interferon activity [48]. This contradictory mechanism highlights the complexity of SARS-Cov-2 infection in the respiratory tract. Ideal survival conditions depend on the interplay of SP-mediated downregulation of ACE2 and interferon-mediated upregulation of ACE2.

The lungs are a conduit through which SARS-Cov-2 accesses the systemic circulation to infect other vital organs and tissues. As the virus travels from the lungs to the rest of the body, it propagates widespread damage and dysfunction by promoting inflammation that culminates in a cytokine storm [49]. This worsens patient outcomes and results in long-term tissue damage [50]. Therefore, it is essential to continue to investigate the pathology and treatments for SARS-Cov-2 infection in the lungs as the primary target of the virus. Notwithstanding, the recognition that ACE2 is also highly expressed in other organs equally warrants examination of the role of this enzyme/receptor in the downstream consequences of SARS-Cov-2 beyond lung infection.

### Not to Be Forgotten: Non-lung Organs Play a Vital Role in COVID-19 Pathology

The neuroinvasive capacity of SARS-CoV-2 has been demonstrated in both humans and animals. Intensive care patients display neurological symptoms such as nausea, headaches, and vomiting [51]. A recent study on SARS-CoV-2 found that 78 out of 214 patients demonstrated infection of the central nervous system (CNS). More severe patients manifested acute cerebrovascular diseases, impaired consciousness, and skeletal muscle injury [52]. Although the exact route SARS-CoV-2 takes to infect the CNS is not known, SARS-CoV-2 is very similar to SARS-CoV and MERS-CoV. Looking at the mechanisms of entry of SARS-CoV and MERS-CoV may provide key insight into the route SARS-CoV-2 takes to infect the CNS. SARS-CoV, like SARS-CoV-2, utilizes the ACE2 receptor for entry to the cell while MERS-CoV enters cells through dipeptidyl peptidase 4 (DPP4). The presence of ACE2 alone is not conclusive of a cell’s vulnerability to infection. Interestingly, human intestinal endothelial cells expressing ACE2 were not infected by SARS-CoV, and hepatocytes, non-detectable-ACE2 expressing cells, were infected by SARS-CoV [53]. SARS-CoV and MERS-CoV were found to infect the brains of patients in the early 2000 s, and most viral material was found in neurons [54]. Transgenic mice inoculated intranasally with SARS-CoV and MERS-CoV displayed a high mortality rate. Mice administered low concentration of MERS-CoV intranasally only displayed viral infection of the CNS, not the lungs, indicating that the cause of death may be directly due to CNS infection. For both SARS-CoV and MERS-CoV infected mice the brain stem was most concentrated with viral particles. Evidence now suggests coronaviruses enter the CNS via synaptic avenues upon infection of peripheral nerve terminals [55]. Swine hemagglutinating encephalomyelitis virus (HEV) particles, a coronavirus, were enclosed in smooth surface vesicles within the axons of mice [55]. Vesicles containing HEV were transported via microtubules to perikaryons and dendrites, where the viral material was later found in high concentrations. Upon replication in these cells, virions were packed in spinule-coated vesicles in the trans-golgi networks. It is hypothesized that coated vesicles mediate endo- and exocytosis of the virus into and out of the CNS. Extracellular virion accumulation may have been the causing factor of dilated synaptic clefts, consequently allowing the trans-synaptic transfer of the coated virus-containing vesicles to other host neurons [55]. This phenomenon must now be explored with SARS-CoV-2 to attenuate brainstem-induced cardiovascular and pulmonary complications. Further elucidation regarding the neuroinvasive capacity of SARS-CoV-2 may provide insight to better mitigate symptoms and prevent infection in humans.

Cardiac involvement in SARS-CoV-2 infection is prevalent among patients [56]. As noted above, the acute disease progression begins by infiltrating the lungs via SP-ACE2 interaction and is then followed by collateral tissue injury and inflammation. Along with inflammation arises vasodilation, endothelial permeability and, leukocyte recruitment, which contribute to the exacerbation of pulmonary injury, hypoxemia, and cardiovascular distress [56]. Severe patients present with systemic inflammation which has the propensity to damage the heart without direct viral infection of the tissue [57]. Direct SARS-CoV-2 infection, hypoxemia, and respiratory failure all also contribute to myocardial injury seen amongst patients. Out of these 3 factors, it remains unclear which is the main contributor to myocardial complications. Cell populations in the heart most vulnerable to infection may be distinguished by expression of ACE2. For instance, myocardial pericytes express ACE2, and infection or disruption caused by inflammation may lead to ischemic injury and disruption of the microvasculature [58]. Biomarkers troponin I and Brain-type natriuretic peptide were both present at augmented levels in severely ill patients [59]. Blood pressure elevation and arrhythmias are common symptoms in patients however this may be due indirectly by systemic inflammation. Acute coronary syndromes are present with SARS-CoV-2 infection. The risk of this instance is significantly increased when an underlying disease is present creating a systemic prothrombotic state [56]. Further investigation on the cause of cardiac complications that come with SARS-CoV-2 must be completed to effectively treat patients and minimalize debilitating injury or death.

Gastrointestinal abnormalities were seen in 2%-10.1% of patients positive for SARS-CoV-2 [60]. Symptoms including diarrhea and vomiting indicate that the virus can interact with cell populations in the gut. The ACE2 receptor is present in the gastrointestinal tract at significant amounts [61]. Recently discovered, viral material was found to be present in infected individuals’ fecal material [62]. The phenomenon of gut-lung communication has been seen previously in other diseases, such as asthma [63]. Further investigation is imperative to possibly mediate SARS-CoV-2 through probiotics or antibiotics [60]. Although only a small percentage of patients experience gastrointestinal symptoms with SARS-CoV-2, this should be taken seriously as it may exacerbate pulmonary complications caused by the virus.

Liver injury is linked to multiple etiologies such as alcohol consumption, toxins, bile duct dysfunction, and viral infections. Epidemiologists reported that 75 out of 148 COVID-19 patients had varying degrees of liver dysfunction [64]. The exact mode of SARS-CoV-2 entry to the liver is unknown, and further research is needed to differentiate whether injuries are due to drug treatments, systemic inflammation caused by pulmonary infection, or viral infiltration of liver tissue [65].

SARS-CoV-2, the cause of viral pneumonia spreading across the globe, can infect many vital organs via SP-ACE2 mediated cellular infection. SP is critical to extrapulmonary organ infection due to its ability to promote viral uptake in these tissues. Furthermore, certain mechanisms in the gastrointestinal tract and heart bolster SP-mediated viral uptake. In the gastrointestinal system, the high concentration of the TMPRSS2 protease enhances the ability of SARS-Cov-2 to enter intestinal epithelial cells [66]. In the heart, pericytes are robust targets of SP binding due to their elevated ACE2 expression, and pericyte infection results in microvascular dysfunction that worsens cardiac symptoms [58] Therefore, infection of non-lung organs may further exacerbate pulmonary infection and other comorbidities such as cardiovascular and cerebrovascular diseases. Establishing effective treatments to mitigate the effect of SARS-CoV-2 on non-lung organs will be very beneficial for sequestering the spread of the virus outside of the lungs, which will likely treat the noted comorbidities.

### Beyond the Horizon: Acute and Chronic Consequences of COVID-19

Although COVID-19 is primarily a respiratory virus, it may have acute and chronic consequences on non-lung organ systems. Delineating how quickly COVID-19 progresses, particularly in patients who develop dire respiratory, cardiovascular, and cerebrovascular complications, is key to understanding the virus’s multi-organ pathology and establishing appropriate treatment.

The incubation period for COVID-19 is approximately 5.1 days. For 97.5% of symptomatic patients, the estimated number of days before symptom onset is 11.5 [67]. Acute respiratory distress syndrome occurs approximately 8 to 9 days after the incubation period [68]. Regarding patients who develop myocardial injury, a median of 18.5 days passed between symptom onset and death [69].COVID-19 patients with cardiovascular complications typically suffer from acute cardiac injury, arrhythmias, and heart failure [70]. According to the National Health Commission of China (NHC), 58% of COVID-19 patients with dire symptoms had hypertension. 44% of these patients suffered from arrhythmia and 25% suffered from heart disease [71]. The NHC stated that 11.8% of patients who died from COVID-19 endured severe heart injury due to high amounts of CTnl or cardiac arrest without having a pre-existing heart condition [72]. Interestingly, the NHC reported that some patients initially showed symptoms such as chest tightness and heart palpitations rather than the common respiratory symptoms suggesting COVID-19 may lead to chronic malady [71]. Some patients previously infected by SARS-CoV later developed chronic cardiovascular damage [71]. For instance, cardiovascular disorders emerged in 44% of 25 SARS-CoV patients 12 years post-infection. Hyperlipidemia progressed in 68% of patients [71]. SARS-CoV spurred a substantial elevation of free fatty acids, lysophosphatidylcholine, lysophosphatidylethanolamine, and phosphatidylglycerol in the blood serum of these patients [71].

The cardiovascular repercussions of COVID-19 infection may be primarily due to the presence of the ACE2 receptor in cardiac tissue. COVID-19 internalization via the ACE2 receptor leads to a loss of the ACE2 peptidase pathway, which can generate a rise in blood pressure, fibrosis, and inflammation [72]. Infected patients have substantially greater angiotensin II (ANG II) levels than healthy individuals, indicating the loss of ACE2 activity [72]. In addition, ACE2 plays a role in the progression of diabetes mellitus and hypertension [71]. Individuals with diabetes and hypertension are at higher risk from COVID-19 because of increased ACE2 expression. Furthermore, COVID-19’s affinity for the ACE2 receptor may have detrimental effects on the cardiovascular system, potentially causing acute and chronic cardiac injury in patients. Although the expression of the ACE2 pathway may be a prominent agent of cardiovascular complications in COVID-19 patients, there may be other signaling pathways that lead to COVID-19-induced cardiac complications. COVID-19 can cause vascular inflammation by spurring the secretion of the following inflammatory factors: troponin, natriuretic peptides, and IL-6 cytokines. Inflammation in the cardiovascular system can lead to diffuse microangiopathy, thrombosis, myocarditis, cardiac arrhythmias, heart failure, and acute coronary syndrome [73]. Regarding the initial 41 confirmed COVID-19 cases in Wuhan, 5 of those patients suffered myocardial affliction shown by the rapid escalation of troponin I level in the cardiac region [71]. COVID-19 can cause myocardial trauma through an increase in inflammatory factors, such as IL-6, lactate dehydrogenase, ferritin, and D-dimer, which indicates the formation of a cytokine storm [69]. The virus can also impair myocardial tissue through hypoxemia and respiratory damage [71]. COVID-19 may also impact the heart directly, inducing myocardial injury through viral myocarditis and stress cardiomyopathy rather than via inflammatory factors [69].

The multi-organ pathology of COVID-19 also includes the cerebrovascular system since the virus may have neurotrophic capabilities [74]. The prospect of complications associated with COVID-19 increases for patients with pre-existing neurological conditions [75]. COVID-19 may manifest in a multitude of acute and chronic neurological diseases, ranging from acute ischemic stroke to encephalitis, Guillain-Barré syndrome, and acute necrotizing hemorrhagic encephalopathy [75].

The incidence of stroke in COVID-19 patients exemplifies the need for vigilance on non-respiratory diseases that may present with devastating symptoms as that produced by the primary lung pathology. Cerebrovascular disease appeared in 5.7% of COVID-19 patients with dire symptoms during the late onset of the virus [74]. On average, stroke occurred 12 days post COVID-19 diagnosis [74]. In Italy, ischemic stroke appeared at a rate of 2.5% in a group of hospitalized patients. This percentage was 5% in China and 3.7% with Dutch COVID-19 patients [75]. The risk of ischemic stroke in COVID-19 patients increases with cardiovascular complications, such as hypotension, heart failure, shock, and arrhythmogenic cardiomyopathy [76]. Cerebral ischemia in COVID-19 patients has been linked to temporary hypercoagulability in some dire cases [77]. Since ACE2 receptors are prominent in the endothelial lining of blood vessels, COVID-19 may spur vascular endothelial damage, increasing the chance of thrombogenesis and cerebrovascular ischemia [77]. COVID-19 may also incite stroke by intensifying inflammation through an increase in D-dimer and CRP [74]. However, the exact mechanism behind COVID-19-induced ischemic stroke is unknown, so further examination is warranted [77].

Along with ischemic stroke, other acute cerebrovascular diseases in the clinical setting of COVID-19 have been explored. In Wuhan, 36% of 214 COVID-19 patients suffered from neurological complications, and 6% of the patients developed acute cerebrovascular disease [77]. COVID-19 may injure the neurological system through direct impairment to particular receptors, secondary hypoxia, cytokine storm generation, and retrograde nervous transport [75]. Starting in the lung, the virus can retrogradely move across neuronal synapses to infiltrate the brainstem, demonstrating how COVID-19 has also caused brainstem-induced cardiovascular and pulmonary complications [75]. COVID-19 can spur glial cell activation and heightened inflammation in the CNS through the escalation of IL-6, IL-12, and IL-15, as well as tumor necrosis factor-alpha (TNF-α) [75]. The virus may also bind to the ACE2 receptors in endothelial cells along the blood-brain barrier, allowing the virus to infiltrate the central nervous system [75]. Moreover, COVID-19 can potentially impair the blood-brain barrier, leading to acute necrotizing encephalopathy [75]. 20% of hospitalized patients developed hypoxic ischemic encephalopathy after the onset of COVID-19 [75]. Additionally, the pathology behind COVID-19-induced encephalitis can be linked to inflammation and edema [75]. PCR has revealed the presence of COVID-19 in cerebrospinal fluid, which may also stimulate encephalitis [74]. In some cases, COVID-19 has incited the development of Guillain-Barré syndrome (GBS), where the body’s immune system attacks nerves, leading to flaccid paralysis. The virus has been associated with 5 GBS cases in Italy and 2 GBS cases in Wuhan [75].

In addition to acute repercussions, COVID-19 infection may have chronic neurological consequences. Although a direct link between COVID-19 and demyelinating disease, such as multiple sclerosis, has not yet been established, the association between human coronaviruses and multiple sclerosis (MS) has been explored. An OC43 coronavirus detection test was conducted on human brain autopsy samples for patients with MS. The MS samples demonstrated a substantially higher level of OC43 than the control group [76]. In addition, MS mice models developed acute encephalomyelitis and a chronic demyelinating condition when infected with the JHMV coronavirus strain [76].

Although the exact mechanism mediating the chronic manifestations of SARS-CoV-2 infection is currently unknown, the interaction of SP and ACE2 contributes significantly. SP-ACE2 binding results in host-cell dysfunction that precipitates the development of these acute and chronic symptoms, including host-cell death. Therefore, the serious long-term repercussions of COVID-19 should be closely monitored in infected patients with high SP and ACE2 expression.

## Choose your Character: Available Therapies in the Fight Against COVID-19

### Human Behavior

Like the current pandemic, the 1918 Spanish flu pandemic disrupted the daily life of the plagued countries [78]. When the first article surrounding this influenza was published in a newspaper in Madrid, only a handful of cases were present. However, this occurred during the season of local holidays in Madrid which entailed people gathering in ballrooms and large parties. These events put people at high risk for exposure and eventually the number of infected individuals was slowly rising [78]. Eventually, towns began to take the pandemic more seriously and various measures were taken. Schools and universities were beginning to shut down for the rest term, yet public gatherings such as church services or cinemas remained open [78]. Public health measures were later adopted including the disinfection of public areas using phenolic oil or creoline which was a popular disinfectant at the time. Cafeterias, churches, tramway wagons, and other public areas were being disinfected. Even mail began to be disinfected. In some cities, the streets were cleaned with a mixture of water and sodium hypochlorite, and spitting was banned. After seeing how quickly the virus was able to spread through public gatherings, Public Health Officials imposed new safety measures for the citizens of Spain [78]. These measures included avoiding public gatherings in closed environments, avoiding any direct contact with those who were ill, and ventilating homes. They also recommended that individuals clean and disinfect their mouth and nostrils with hydrogen peroxide [78].

COVID-19 has disrupted the lives of people worldwide in an eerily similar fashion. The virus originated in Wuhan, China with the first case documented in December 2019. After only a few months, the virus began spreading worldwide. By March, COVID-19 was declared a pandemic by the World Health Organization (WHO). Many schools and universities shut down beginning in the middle of March and transitioned into virtual classes taken online at home. Adults have also been quarantined at their homes with jobs and businesses shutting down around the same time as schools in March with an exception to essential workers such as healthcare workers and grocery store workers. Having individuals stay home largely limits the number of people gathering in public. These measures, more commonly known to the public as social distancing, are implemented to reduce the number of interactions between individuals. This prevents individuals who are infected with the virus but have not been diagnosed with COVID-19 or are asymptomatic to interact with healthy individuals. COVID-19 is transmitted by one individual to another when in close proximity [79]. Social distancing aims to reduce transmission of the virus by physically separating individuals. This includes the closure of office buildings, a ban on large public gatherings, and the separation of individuals in public [79]. Guidelines created by the CDC recommend that people avoid any close contact with individuals who are sick and maintain 6 feet of distance from others. Another method used to prevent the spread of COVID-19 is isolation. Patients who are diagnosed with COVID-19 or are suspected to have been exposed are isolated in hospitals to prevent the transmission of the disease to non-infected individuals [79]. Patients are typically isolated for the full duration of the virus’s incubation period to ensure that they are no longer infected when leaving isolation. Isolation can occur individually or in groups. Individuals have also started to self-isolate themselves at their own homes. When individuals begin to feel ill, they refrain from any public activity such as their job and will isolate themselves at home. Individuals also practice this when they have been exposed to another individual who has COVID-19 [79]. The CDC also recommends avoiding any mass gatherings and crowded areas and covering the mouth and nose with a mask or face cover when around others.

Quarantine is an old yet effective tool in controlling disease outbreaks. This practice was commonly employed in fourteenth-century Italy when ships coming from a plague-infected port arrived at a port in Italy [79]. The ships were required to anchor and wait for 40 days before passengers were allowed to disembark. These 40 days allowed for asymptomatic passengers to begin showing symptoms and be identified. This allowed for only healthy passengers to be allowed off the ship while infected individuals were sent to get treatment. This also prevented the asymptomatic passengers from spreading the disease to people in the port city.

This quarantine method was also implemented during the SARS epidemic in 2003. This measure was thought to be successful and effective [79]. Quarantine means restricting the movement of individuals who have been exposed to a disease but are not ill or are simply asymptomatic. By restricting the movement of these individuals, the number of interactions they have with other individuals is decreased which is crucial if they are infected with a disease. During the quarantine period, individuals are carefully monitored for signs of any symptoms [79]. Quarantine methods have been used during the COVID-19 pandemic, especially on those individuals who have traveled to countries with high rates of COVID-19. Many travelers who returned from a country with high COVID-19 rates such as China were subjected to a mandatory 2-week quarantine. Some countries used isolation tents to conduct the 2-week quarantine while others simply strongly recommended individuals to self-isolate at home.

Quantitative assessments of the effectiveness of quarantine may provide empirical evidence for the recommended guidelines. The Susceptible-Exposed-Infectious-Recovered (SEIR) model shows the speed at which individuals move from the susceptible state onto the exposed, infectious, and recovered state [80]. Preventive measures taken in China such as travel bans, home quarantine, and extended vacations have been successful in decreasing the transmission of infection. There were decreases in the transmission of COVID-19 after these measures, however, they are only effective if the isolation period is long enough [80].

Good hygiene also plays a key role in preventing the spread of disease. Hand hygiene has been proven to be especially significant. Appropriate handwashing can break the transmission cycle and reduce the risk of spreading disease remarkably [81]. Health officials recommend frequent handwashing and avoiding touching the nose, mouth, or eyes with unwashed hands to prevent the spread of disease. An equally essential hygiene protocol requires cleaning and disinfecting any surface that is touched daily like a phone or a light switch. Additionally, mask-wearing has been recommended by the Center for Disease Control (CDC) to block pathogens from entering the respiratory tract. It is recommended to wear a mask whenever in public and in direct contact with other individuals regardless of whether they are ill or not. Stronger masks such as N95 masks can block 91% of pathogens [81]. While wearing a mask has been recommended by health officials, the need for better equipment to avoid the spread of the virus intranasally may require innovations in the use of air purifiers and nasal protective devices. Finally, it is critical to monitor one’s health and be alert for any possible symptoms of COVID-19. For those experiencing the symptoms of COVID-19, urgent medical care is highly recommended. If the COVID-19 test comes back positive, the CDC recommends staying at home except to receive medical care. It is essential to stay away from others while ill and to disinfect any surfaces that come into contact with the patient. The best methods to stay proactive about COVID-19 are to maintain good hygiene and follow the CDC behavioral guidelines. Washing hands frequently, remembering to wear a face mask in public areas, and practicing social distancing guidelines are all efficient ways to protect from COVID-19.

### Vaccines

When the Spanish flu began spreading to other countries such as the U.S., finding a vaccine became a tantamount goal for scientists and clinicians alike. During the peak of this epidemic Spanish flu “vaccines” were developed and distributed throughout the U.S. Health officials informed the public that these vaccines would protect from the influenza. However, the specific cause of influenza was yet to be discovered [83]. These vaccines targeted multiple types of bacteria that had been isolated from victims of the Spanish flu, suggesting that influenza was a bacterial disease. These Spanish Flu vaccines were widely used but still had little to no supporting evidence for their benefits. Only a few attempts to test their effectiveness were made once the vaccines were distributed [82]. Eventually, the Surgeon General, American Public Health Association, and editors of the Journal of the American Medical Association made a public statement that there was currently no serum or any means of curing influenza and that there was no vaccine to prevent it. They informed the public that all claims made by newspapers and other sources were unofficial and experimental. This editorial warned health officials and physicians to stop making false promises without evidence to support them. Without knowing the causative agent of the Spanish flu there was no evidence to suggest the effectiveness of a vaccine [82]. The scientific community was failing to find the cause of the influenza which led researchers to believe that influenza was a viral disease. Virology was still in its early stages during this time. However, by the 1930 s there was an improvement in the process of isolating and identifying pathogens [84]. These research advances aided in the search for the Spanish Flu vaccine.

The Spanish flu vaccine was not developed until 2005, more than 80 years after the beginning of the outbreak. The virus causing the Spanish Flu, known as the H1N1 virus, is considered an avian influenza virus that underwent multiple mutations. Another avian influenza virus known as H5N1 began to infect humans and had a mortality rate of approximately 60% [83]. Although human to human transmission was not possible at the time of this bird flu pandemic, researchers were worried that this virus could mutate and have the capability to transfer from human to human posing a dangerous threat to public health. The live attenuated influenza vaccine elicits an immune response that triggers the production of chemokines and cytokines associated with T-cell activation that clears the virus rapidly [83].

Currently, there is no vaccine that prevents COVID-19. While human behaviors, including social distancing and proper hygiene that plagued the Spanish flu, remain a challenge in battling COVID-19, there is much clamor for evidence-based and science-supported treatments in the current pandemic. In particular, databases for registered clinical trials have been established allowing a much better translation of safe and effective approaches for COVID-19. The genome of the SARS-CoV-2 was sequenced in mid-January triggering researchers around the world to begin developing a vaccine [84]. The first COVID-19 vaccine candidate began human clinical trial in mid-March (ClinicalTrials.gov: NCT04283461). As of April 8, 2020, there were 115 global COVID-19 vaccine candidates, 73 of which were currently at preclinical stages [84]. Some of the more advanced candidates include mRNA-1273 developed by Moderna (NTC04283461), Ad5-nCoV developed by CanSino Biologicals (NCT04313127), and INO-4800 developed by Inovio (NCT04336410). These candidates have moved into clinical development. The vaccine development for COVID-19 has included a range of technology platforms. These include nucleic acid, recombinant protein, viral vector, among others. Platforms based on DNA or mRNA such as the vaccine being developed by Moderna offer flexibility in terms of antigen manipulation as well as a potential for speed. Moderna began clinical testing only 2 months after sequence identification. Platforms based on viral vectors offer a high level of protein expression, induce strong immune responses, and offer long term stability. Vaccines based on recombinant proteins can take advantage of other recombinant protein-based vaccines from other diseases giving them a large-scale production capacity (NCT04568988). Public information regarding the specific SARS-CoV-2 antigen used in vaccines is very limited. Many candidates who have revealed information aim to induce neutralizing antibodies against the viral SP. Targeting SP is crucial to developing a potent vaccine that blocks the virus from binding to ACE2 and entering host cells, thus preventing infection from occurring [84]. Polyclonal murine antibodies against SP already effectively inhibit SARS-Cov-2 uptake in mice [13]. Knowledge of the exact molecular structure of SP will facilitate the development of human antibody-based vaccines that potently inhibit SP-mediated SARS-Cov-2 cell entry.

Most of the COVID-19 vaccines that are being developed are in North America, while there is also development activity in China, other parts of Asia, Australia, and Europe. The global vaccine effort has been exceptional in terms of scale and speed. Projects indicate a vaccine may be available as soon as early 2021. This would be a massive change from traditional vaccine development which takes on average over 10 years. Even the accelerated development of the Ebola vaccine took 5 years [84]. To assess the efficacy of these vaccines, COVID-19-specific animal models are being developed. These include ACE2-transgenic mice, hamsters, ferrets, and non-human primates [84]. SARS-CoV-2 vaccine development poses some obstacles. It is crucial to optimize antigen design to ensure we receive an optimal immune response. Some researchers argue the best approach is targeting the full-length protein, while others believe that targeting only the receptor-binding domain is more efficient [85]. Researchers are also worried about exacerbating lung diseases as a result of antibody-dependent enhancement as seen previously with SARS and MERS vaccine candidates. This effect may be associated with a type 2 helper T-cell response. This is why it is critical to test all vaccines with a proper animal model and to safely monitor all clinical trials [85]. Adjuvants may be required to generate a sufficient immune response. Adjuvants triggering a Th1 response and showing a high neutralizing antibody response are more suited to be protective (NCT04368988). Although we can infer the duration of protection received from a vaccine by looking at vaccine experience with SARS and MERS, we do not know the duration of protection from a COVID-19 vaccine. More research is required to find the duration of immunity as well if vaccines will be potent in single or multiple doses [85]. Vaccine development has been moving at a rapid pace since the genetic sequence for SARS-CoV-2 was released in January. Moderna’s mRNA-based candidate entered clinical trials only 10 short weeks after the sequence was published. Since then many other developers have begun the clinical trial phase (NCT04283461). Some candidates have even begun to manufacture additional materials for clinical trials in phase 2 studies. After phase 2, commercial manufacturing will take place. Facilities capable of producing large quantities of product will need to be identified, proper technology will need to be transferred, and manufacturing processes will need to be adapted. All of the research and development procedures need to be performed without knowing the clinical efficacy of the vaccine candidate [85]. In situations like this COVID-19 outbreak, it is inefficient to conduct randomized and controlled trials with placebo groups. Although this approach is feasible, it is not nearly as fast, and results are often harder to interpret [85]. A possible method moving forward could be to test several different vaccines simultaneously in a trial designed using one shared control group. This allows more participants to receive an active vaccine. This method is advantageous in terms of the number of participants receiving the vaccine and the speed of the trial, however, developers often avoid trials that generate comparative data [85]. Currently, most of the leading vaccine developments are still in phase I of clinical trials. Further data and investigation on these possible vaccines are necessary to assess the efficacy of these vaccines. If any of these vaccines prove to be viable in their later phases, developers will be able to manufacture vaccines as early as the start of next year.

### Antiviral Drugs

In response to the influenza virus, the Global Influenza Surveillance Network was established in 1947 and is responsible for the continuous monitoring of antiviral drugs for clinical treatment [86]. Two antiviral compounds, M2 ion channel blockers (adamantanes) and NA inhibitors are the current options available to combat infection and counteract the spread of viruses. Adamantanes are commonly used to inhibit IAV virus replication and block entry and NA inhibitors block the release of virions after budding from the host cell. However, the rapid emergence of drug-resistant viral strains has placed limitations on the use of NA inhibitors and M2 blockers [87]. Therefore, more initiatives should be put forth for the development of new drugs and clinical assessment. The antiviral medication Amantadine is no longer recommended due to the increase in resistance to H1N1 and H3N2 and it only inhibits influenza A. Oseltamivir is continuously used, but the recent development of new target-oriented drugs such as zanamivir, favipiravir, baloxavir, and pimodivir sheds new light on the treatment for influenza.

There is currently no proven effective antiviral treatment for COVID-19. However, many antiviral treatments have demonstrated efficacy for the treatment of COVID-19 including lopinavir and ritonavir, chloroquine, and hydroxychloroquine that may help to alleviate symptoms in patients. With over 300 clinical trials currently testing various antiviral drugs, treatment has focused on the uses of chloroquine, hydroxychloroquine, lopinavir/ritonavir, favipiravir, remdesivir, and oseltamivir. The focus has been primarily on repurposing the available therapeutic drugs to treat patients with the SARS-CoV-2 infection. Hydroxychloroquine and chloroquine are antiviral drugs that possess similar chemical structures and have been commonly used for the treatment of rheumatoid arthritis and malaria [88]. Their mechanism and action involve targeting lysosome to control graft-versus-host disease in humans. Chloroquine can inhibit the entry of SARS-CoV-2 and prevent virus-host cell fusion by interfering with glycosylation of the ACE2 receptor and its binding with SP. This suggests the potential use of chloroquine in the early stages of infection before the virus can reduce ACE2 expression and activity [89]. Both hydroxychloroquine and chloroquine can inhibit certain cellular functions and molecular pathways involved in immune activation and are both being tested in *in vitro* studies for effectiveness [88]. The U.S. Food and Drug Administration (FDA) has authorized the use of chloroquine and hydroxychloroquine for emergency treatment of COVID-19 and has the potential to decrease symptoms and progression of the disease. However, both drugs have been associated with cardiac risks in patients and more evidence is required to determine the safety and effectiveness of these medications in treating COVID-19.

Another antiviral drug being explored for the treatment of COVID-19 is lopinavir/ritonavir (LPV/r). Lopinavir is a protease inhibitor and ritonavir is a booster and they are commonly used to treat HIV infection in combination. *In vitro* studies have demonstrated the ability of lopinavir in inhibiting SARS-CoV, reducing symptoms such as diarrhea and recurrence of fever, and lowering the risk of acute respiratory distress syndrome with LPV/r and ribavirin [88]. The use of LPV/r for treatment of COVID-19 has proven effective in combined therapy for reducing serious complications such as acute kidney injury and secondary infections [90]. A combination of lopinavir and ritonavir has significantly improved the clinical condition of SARS-CoV patients and serves as a viable treatment option in COVID-19 infections [91].

Favipiravir (brand name Avigan) has been developed and used for the treatment of avian influenza or other types of influenza that are resistant to NA inhibitors. Favipiravir inhibits the RNA-dependent RNA polymerase of RNA viruses such as Ebola, influenza, and norovirus and induces lethal RNA transversion mutations to produce a nonviable virus phenotype [90]. Favipiravir is converted to an active phosphoribosylated form in cells that can be recognized by viral RNA polymerase and inhibiting RNA polymerase activity [92]. Repurposed favipiravircan be used as an experimental agent against single-strand RNA virus SARS-Cov-2 and clinical trials have demonstrated that patients treated with favipiravir have a superior recovery rate [89]. In mild-moderate COVID-19 patients, favipiravir reduced the time of fever reduction and cough relief, but concerns exist relating to adverse effects and not enough knowledge has been produced to recommend favipiravir as an effective treatment [88].

Remdesivir is another novel antiviral drug that was originally developed for the treatment of the disease Ebola. Remdesivir can inhibit viral RNA polymerases and has widespread activity usage against filoviruses and coronaviruses. *In vitro* testing has developed to demonstrate remdesivir activity against SARS-Cov-2 and demonstrates clinical improvement. In animal experiments, the drug has proven to reduce viral load in lung tissue of mice with MERS-CoV, improve lung function, and alleviate the lung damage. Additionally, Remdesivir yields promising results for COVID-19 patients in recovering from pneumonia. In a study of patients using the drug in the United States, 70% of patients had improvement in regards to oxygen requirements and were extubated from mechanical ventilation [89]. Currently, there are four clinical trials in the United States, and two additional trials in China registered on ClinicalTrials.gov [88]. Remdesivir serves as a promising therapeutic treatment for COVID-19.

Additionally, oseltamivir (Tamiflu), which is approved for the treatment of influenza A and B, is used to target the neuraminidase distributed on the surface of the influenza virus to inhibit the spread throughout the body. Several clinical trials are studying the effectiveness of oseltamivir against COVD-19 and also in combinations with chloroquine and favipiravir. However, a study in Wuhan reported no positive outcomes after administering oseltamivir and it does not serve as a recommended treatment for COVID-19 [88].

A potential target for drug development for COVID-19 also involves inhibition of ACE2, the host cell receptor for the S protein of SARS-CoV-2 that is primed by TMPRSS2 protease and may prevent the entry of the virus. The antimalarial drugs, chloroquine, and hydroxychloroquine have been shown to inhibit terminal phosphorylation of ACE2 and serve as candidate drugs against the COVID-19 disease [90]. Chloroquine has inhibition activity against SARS-CoV-2 at relatively high doses but includes a potential risk of side effects. Additionally, the triple combination of cepharanthine/selamectin/mefloquine hydrochloride serves as a candidate drug combination against the SARS-CoV-2 infection with access through ACE2 [90]. The ability to weaken the binding of the virus to ACE2 may prevent entry and replication of the virus and can be blocked by experimental and established drugs. Further studies are required to determine the benefits of such medications and their therapeutic potential in inhibiting the binding of the virus to ACE2.

With over 300 clinical trials targeting various antiviral medications for the treatment of COVID-19, there are still no final verified antivirals specific to COVID-19. Further testing is still needed to explore the efficacy and safety of these antiviral drugs. Research regarding SARS-CoV-2 has demonstrated the potential of repurposing drugs with appropriate pharmacological effects and therapeutic efficiencies in the treatment of COVID-19 patients [89]. The antiviral drugs being explored including hydroxychloroquine, chloroquine, remdesivir, favipiravir, and lopinavir and ritonavir may be able to limit the spread of the virus and reduce morbidity and mortality caused by the COVID-19 pandemic. Since a vaccine is still not available, more research is required to approve antiviral drugs against SARS-Cov-2. From recent clinical trials, the antiviral drugs remdesivir, favipivir, and lopinavir and ritonavir can be administered for the treatment of severe COVID-19 pneumonia and to lower the mortality rate of the disease [90]. To this end, antiviral treatment of COVID-19 is promising, with several potential drug candidates that demonstrate the capacity to perturb the growth and development by interfering with SP and ACE2. Azithromycin targets SP directly, preventing viral uptake, while hydroxychloroquine interferes with ACE2 terminal glycosylation, thus weakening SP-ACE2 binding [93, 94]. Overall, the repurposing of available drugs for immediate use of treatment for COVID-19 could improve the currently available clinical management.

### Convalescent Plasma

Plasma therapy is the process of extracting therapeutic molecules, such as antibodies and plasma, from the immunized or recovering individual and transferring the extracted molecule to ill individuals [95]. This type of regenerative medicine was first developed to battle the Spanish flu during the early 20th century [96, 97]. During the Spanish flu pandemic, transfusion of blood from influenza survivors significantly decreased mortality rates [97]. Another notable discovery was made during a latter viral outbreak. During the arenavirus outbreak, victims often experienced severe hemorrhage [96]. *In vivo* studies during this outbreak highlighted the protective abilities of plasma therapy. Specifically, transfusing blood from immune species into healthy species decreased the severity of the symptoms. These discoveries opened the possibility of utilizing plasma therapy to battle against viral outbreaks. Tremendous advancements were made in the molecular techniques and production of human immunoglobin during the years after each subsequent pandemic or epidemic, including measles, HIV/AIDS, and 2003 SARS-CoV. After several past experiences with viral outbreaks worldwide, there was one constant observation: plasma therapy must be applied as soon as possible after the first onset of disease. Clinical trials have reported that healthy and infected patients who received plasma therapy early displayed a decrease in symptom severity and mortality rate [96]. Based on previous pandemics and discoveries on plasma therapy, if plasma therapy became an available treatment option for COVID-19 patients, convalescent plasma therapy should be applied at the earliest, either as a preventive measure or early therapeutic option, to maximize the efficiency of the treatment.

Plasma therapy may be used to harvest antibodies from individuals who have been exposed to the SARS-Cov-2 to treat COVID-19 patients with pulmonary, neural, or cardiovascular dysfunctions. The transplanted antibodies may bind to SP or ACE2 sites, preventing the coronavirus from fusing with the cell membrane and injecting its viral genome. By blocking this crucial entry point, inflammatory symptoms and infections significantly decreased compared to patients without antibodies [98]. This occurs due to the correlation between inflammatory symptoms and infections and the expression of ACE2. If no blockage is present, the virus binds to pulmonary ACE2 and transfers its viral genome into the cell, causing cell death [99]. The increased cell mortality and loss of ACE2 expression stimulate inflammatory and immune activity [99]. Inflammatory response and overreaction of the immune system to the increased infection and cell death would further cause pulmonary damage, possibly worsening respiratory distress, post-viral infection, and severe acute respiratory failure [99, 100]. Conversely, blocking ACE2 or viral SP with antibodies would preserve ACE2 expression and lower cell mortality, decreasing inflammatory symptoms and infections. Viral infections may also damage neural and cardiovascular cells, decreasing ACE2 expression in the brain and heart, respectively [99, 100]. Similar to pulmonary damage, neural and cardiovascular cell mortality induces inflammatory activity, further causing structural damage and cognitive and cardiovascular dysfunctions [99, 100]. On the other hand, blocking ACE2 or SP via antibodies prevents cell death from viral infection, which consequently preserves ACE2 expression and increases anti-inflammatory activity.

Based on previously mentioned research advancements in plasma therapy, its utility for COVID-19 patients has reached clinical testing. In addition to 152 publications of review and commentary articles on convalescent plasma therapy for COVID-19, there are 173 ongoing clinical trials that are investigating the use of convalescent plasma for COVID-19. Small clinical studies conducted in the US and East Asian countries have displayed an increase in antibody titers and a decrease in the viral activity of plasma-treated patients [101–105]. The viral load in COVID-19 patients was nearly undetectable and assumed to be eliminated after treatment [102, 104]. Despite different dosage of plasma, ranging from 200 mL to 500 mL, no findings on adverse events of convalescent plasma therapy were reported [101–103]. Additionally, significant recovery in respiratory functions was demonstrated seven days after convalescent plasma infusion [102]. Infusion of convalescent plasma also stabilized the health conditions of critically ill COVID-19 patients, removing the need for ventilators over time [101]. Patients who fully recovered after receiving convalescent plasma therapy were discharged from hospitals [103]. Current evidence favors the effective use of convalescent therapy for COVID-19. To further encourage the idea of plasma therapy as a possible COVID-19 treatment, direct comparisons of protocols, safety, and efficacy in ongoing or proposed clinical trials should be made to assess the potential of convalescent plasma for COVID-19.

In one clinical trial, 10 Chinese patients with severe cases of COVID-19 received convalescent plasma treatment after approximately 16 days of contraction [102]. 200-mL convalescent plasma was transfused into each patient. Patients were able to tolerate this level of dosage without displaying any symptoms [102]. Clinical symptoms significantly improved in all patients 3 days after receiving treatment followed by no traces of SARS-CoV-2 after one week. Improvement in pulmonary functions, such as oxygen saturation and lymphocyte counts, due to antibodies obtained from the treatment highlight decreased inflammation and hyperactivity of the immune system [102]. In another clinical study in South Korea, two patients received convalescent plasma treatment. Similar to the previously described study, both patients were diagnosed with severe pneumonia and acute respiratory distress syndrome [101]. Both patients received a total of 500 mL dose of convalescent plasma, which was administered twice in 12-hour intervals. Patients received treatment at different times: one receiving 22 days after onset of symptoms and the other after 7 days [101]. No adverse effects of the transfusion were present in either patient. Both patients demonstrated a significant recovery in respiratory functions and levels of lymphocytes increased almost immediately after plasma therapy [101].

Both clinical studies presented findings that parallel previous discoveries from earlier pandemics and studies regarding convalescent plasma treatment. Earlier treatment after exposure to SARS-CoV-2 was more effective compared to later treatments [102]. As seen in previous studies, inflammatory activity decreased and normal pulmonary and respiratory functions returned after convalescent plasma therapy [101, 102]. Additionally, plasma therapy does not present any detrimental or adverse reactions to patients even when administered large dosages [101, 102]. These results suggest that plasma therapy is safe and effective for COVID-19 patients, especially when treated early. However, some limitations are present in the two clinical trials. Both clinical trials had a small number of subjects. Furthermore, some patients received other sources of standard care while also receiving plasma therapy, such as antiviral treatment [102]. Indeed, this opens the possibility of antiviral treatment contributing to the recovery of the patients. However, this also raises uncertainty regarding the true therapeutic effects of plasma therapy in human subjects. Additionally, some patients were treated much later after exposure to SARS-CoV-2 compared to other patients [101, 102]. For patients who received plasma treatment late, it is difficult to determine whether their recovery was due to convalescent plasma or natural recovery. The varying times of treatment along with limited human trials limit the potential of convalescent plasma infusion. Future clinical studies should increase the number of test subjects and apply plasma therapy as early as the first onset of symptoms.

### Stem Cells

As noted above, plasma therapy involves the use of convalescent plasma to transfer antibodies and boost the patient’s anti-inflammatory response against COVID-19 [106, 107]. With the advancements in purification technology over the past several decades, human immunoglobulin for intravenous use have been used as the second line of passive immunotherapies against these diseases using manufactured plasma-derived immunoglobulins (IG) [106, 107]. In addition to immunoglobulins, the blood contains a heterogeneous population of cells, one of which are stem cells, that may confer therapeutic benefits. If the stem cells stand as active components rendering the therapeutic inflammatory response in convalescent plasma, then isolating these stem cells, as opposed to the whole plasma treatment, may afford equally or more robust functional outcomes in COVID-19 patients.

Stem cell therapy stands as the prominent regenerative approach in a wide range of diseases, which interestingly include COVID-19 primary symptoms (i.e., lung disease) and co-morbid diseases such as but not limited to diabetes, cardiovascular and cerebrovascular disorders [108]. Besides their regenerative capabilities, stem cells release immune system modulators which highlight their applicability in COVID-19’s aberrant immune and inflammatory response. To this end, stem cells can impart protection by attenuating inflammation and are being explored as treatment options for COVID-19 in clinical trials (Table 1). Preclinical studies have consistently shown the therapeutic effects of stem cell therapy on animal models of diseases plagued with impaired immune and inflammatory reactions [112–114], and the benefits are thought to be due to the release of modulators that attenuate the deleterious immune and inflammatory response that leads to secondary cell death.

**Table 1:** Novel clinical trials assessing the safety and efficacy of stem-cell based therapeutics in COVID-19 patients

Clinical Trial	Cell type	Phase of trial	Significance
NCT04331613	Human Embryonic Stem Cells (CAStem)	Phase II	CAStem cells will be intravenously injected into patients with or without acute respiratory distress syndrome (ARDS) induced by COVID-19. Patients will receive doses of either 3, 5 or 10 million cells/kg, and dose escalation will ensue if initial cell infusion proves to be safe. Adverse events, mortality rate, time it takes for RT-PCR to be negative for SARS-CoV-2, and changes in blood oxygen levels will be assessed. In addition, levels of IL-1 beta, IL-2, IL-6, and IL8 will be evaluated to further elucidate CAStem efficacy.
Leng et al. [109]	Mesenchymal Stem Cells (MSCs)	Complete	Post intravenous MSC administration, COVID-19 patients demonstrated ameliorated pulmonary function two days after treatment, upregulation of peripheral lymphocytes, reduction in C-reactive protein, and elimination of CXCR3+CD4+ T cells, CXCR3+CD8+ T cells, and CXCR3+ NK cells 3-6 days following treatment.
NCT04313322	Wharton-Jelly derived mesenchymal stem cells (WJ-MSCs)	Phase I	COVID-19 patients will be administered WJ-MSCs intravenously at a dosage of 1X10e6/kg. They will be given three doses three days apart. Clinical improvement will be evaluated over three weeks in addition to the conduction of a CT scan and RT-PCR for viral RNA.
NCT04473170	Autologous non-hematopoietic peripheral blood stem cells (NHPBSCs).	Phase II	Survival rate and clinical improvements for COVID-19 patients are to be monitored after treatment with (NHPBSCs). Patient immune profile will be evaluated, measuring levels of immune biomarkers, such as CD3, CD4, CD8. Number of acute phase proteins and Inflammatory markers (e.g. CRP, ESR, IL-6) will also be examined.
Ye et al. [110]	Allogeneic human dental pulp stem cells (DPSCs)	Phase II	20 patients with severe pneumonia induced by COVID-19 were administered intravenous DPSCs at a dosage of 3.0x107 human DPSCs in 30ml saline solution. The trial began on April 6th and will continue until December 31st. Neutrophil, T lymphocyte, B lymphocyte, natural killer cell, and macrophage levels, along with alterations in serum cytokine levels (IL-1 β, IL-2, TNF-a, ITN-γ, IL-4, IL-6, IL -10) will be examined.
NCT04366323	Allogeneic and expanded adipose tissue-derived mesenchymal stem cells	Phase II	Allogeneic and expanded adipose tissue-derived MSCs administered in two doses (80 million cells) to patients with severe pneumonia caused by SARS-CoV-2 infection. Safety and efficacy will be measured through the frequency of adverse events and mortality rate.
NCT04346368	Bone-marrow derived mesenchymal stem cells	Phase II	BM-MSCs will be delivered intravenously at a dose of 1*10E6 /kg to severe COVID-19 patients. Safety and efficacy of cell-based treatment assessed through clinical symptom amelioration, frequency of adverse events, and mortality rate. Improvement of pneumonia will be evaluated through alterations in PaO2/FiO2 ratio and CT scan. Viral density, changes in CD4+, CD8+ cells, and cytokine levels will also be analyzed.
NCT04416139	Umbilical cord derived mesenchymal stem cells (UC-MSCs)	Phase II	Patients with acute respiratory distress syndrome caused by COVID-19 will be treated with intravenous UC-MSCs at a dose 1 million xKg. Patient improvement will be evaluated over three weeks, along with the assessment of the immune profile, investigating the stem cells’ effect on the cytokine storm. Changes in TNFa, IL-10, IL-1, IL-6, and IL-7 cytokine levels in plasma will be noted.
NCT04437823	Umbilical cord derived mesenchymal stem cells	Phase II	COVID-19 patients will receive intravenous treatment of UC-MSCs at a dose of 5 x 10^5 cells/Kg on days 1, 3, 5. The frequency of adverse events and mortality rate will be assessed to determine clinical efficacy, along with the conductance of CT scans, PCR tests, and sequential organ failure assessment (SOFA).
Sengupta et al. [111]	Bone-marrow derived mesenchymal stem cells	Complete	Exosomes (ExoFlo™) isolated from allogeneic BM-MSCs were administered intravenously (15mL) to 24 severe SARS-CoV-2 patients. The clinical condition and oxygenation state of patients were significantly ameliorated and an 83% survival rate was detected. The average amount of neutrophils decreased and mean numbers of CD3+, CD4+, and CD8+ lymphocytes were upregulated.
NCT04315987	NestaCell® (MSC)	Phase II	COVID-19 patients will be treated with intravenous NestaCell® at a dose of 2x10^7 cells on days 1, 3, 5 and 7. Mortality rate and respiratory improvement will be assessed over 10 days. Oxygen saturation will be measured through Hypoxia status and PaO2/FiO2 ratio. CD4+ and CD8+ T cell levels will also be evaluated to determine the immune profile.
NCT04429763	Umbilical cord-derived mesenchymal stem cells	Phase II	Severe COVID-19 patients will be treated with single dose of UC-MSCs (1*10^6 cells/Kg). Over 4 weeks, mortality rate and clinical decline will be assessed.
NCT04457609	Umbilical cord-derived mesenchymal stem cells	Phase I	In conjunction with standardized treatment (Oseltamivir + Azithromycin), patients will be treated with intravenous UC-MSCs at a dose of 1x10^6 cells/Kg. Clinical amelioration will be assessed through the presence of dyspnea and sputum, fever, ventilation necessity, monitoring of blood pressure, heart rate and respiratory rate, and oxygen saturation. CXCR3, CD4, CD8, and CD56 cell counts, along with IL-6 and IL-10 levels will be analyzed to distinguish the anti-inflammatory capabilities of UC-MSCs.
NCT04486001	Adipose-derived allogeneic mesenchymal stem cells	Phase I	Adipose-derived allogeneic MSCs will be intravenously delivered to COVID-19 patients. Clinical improvement following stem cell treatment will be assessed via frequency of adverse incidents, mortality rate, the number of ventilator and ICU free days, total hospital and ICU days, and improvement in oxygenation.
NCT04390152	Wharton-Jelly mesenchymal stem cells	Phase II	Patients will receive two doses of WJ-MSCs (50*10e6 cells) along with standardized treatment of hydroxychloroquine and Lopinavir/Ritonavir or Azithromycin and ventilator support. Mortality rate, sequential organ failure assessment (SOFA), and frequency of adverse events will be evaluated. In addition, WJ-MSC efficacy against the cytokine storm will be assessed by monitoring levels of IL-6, IL-8, IL-10, and TNF alpha.

Prominent sources of stem cells for regenerative therapy are embryonic stem cells (ESCs), fetal stem cells, induced pluripotent stem cells (iPSC), and adult stem cells. Pluripotent ESCs generated from the blastocyst can differentiate into all three germ layers [110]. Their great differential ability elucidates their therapeutic potential for regenerative therapy, as they can evolve into specialized cells [115]. However, ethical concerns surrounding the destruction of embryos and high risk for tumorigenicity limit the use of ESCs in clinical applications. Fetal stem cells are produced from fetal tissues, such as the placenta, amniotic fluid, and fetal membranes [116]. They have great proliferative capabilities, can differentiate into a variety of progenitor cells, and are less likely to spur immune rejection [117]. iPSCs are genetically reprogrammed somatic cells that behave similarly to ESCs [110]. They show promise in the treatment of degenerative disorders due to their self-renewal and differentiative capabilities [118]. However, they are likely to be rejected by the host and have the highest tumorigenicity of other stem cell types. Adult stem cells are generated from adult tissue and include mesenchymal stem cells (MSCs), hematopoietic stem cells, and resident adult stem cells [119]. They show significant therapeutic potential for damaged tissue repair as they can be engineered to differentiate into specialized cells, replacing impaired cells, as well as secrete anti-inflammatory agents [110, 119]. MSCs derived from bone marrow and the human umbilical cord demonstrate significant therapeutic efficacy for regenerative therapy and have a long track record of safety in hematologic disorders [120]. MSCs carry significant restorative capabilities, as they can differentiate into specialized cells like chondrocytes, adipocytes, and osteoblasts and secrete paracrine and autocrine factors that promote tissue rehabilitation and ameliorate inflammation [121]. Clinical trials using MSCs to treat hematologic disorders have confirmed their safety profile, expediting their application in non-hematological disorders, and the use of MSCs for brain disorders associated with a destructive immune response like stroke and TBI have reached clinical trials with promising results [122, 123].

Because MSCs possess the ability to regulate the immune and inflammatory activity, MSC therapy can potentially be used to treat COVID-19. As previously described, the intermolecular interaction between the viral SP and human ACE2 initiates the infection of host cells [124]. Once the virus enters the host, macrophages of the immune system detect and bind to the foreign molecule to fend off the virus, recruiting proteins and initiating the immune cascade in the process [39]. The viral-induced immune cascade causes extensive tracheobronchial inflammation, further causing pulmonary damage in COVID-19 patients and promoting SARS and ARDS symptoms, such as pulmonary edema, hypoxia, respiratory distress, and lung damage [40]. Furthermore, replicated SARS-COV-2 utilizes the lungs to circulate within the patient’s body. Similar to the pathology of the pulmonary infection, SARS-CoV-2 can infiltrate and infect ACE2-harboring cardiac or neural tissue. The viral activity prompts an increased immune response, leading to inflammation and further causing damage in the heart or brain [36, 39]. Since COVID-19 causes pulmonary damage as well as cardiovascular and cerebrovascular infection by overreacting the immune system and increasing inflammation, MSC transplantation may be a suitable approach against COVID-19.

In addition to its anti-immune and anti-inflammatory mechanisms, MSCs may possess the ability to interfere with viral docking via ACE2-SP interaction. As previously discussed, MSCs regulate immune and inflammatory activity by inhibiting inflammatory factors and cytokine storms. MSCs are able to bind to various surface receptors to activate these mechanisms, such as IL-1R, TNFI, and IIR [125]. Due to its immune-sensing adaptability, MSCs may also bind to ACE2, an entry receptor for SARS-CoV-2. If MSCs possess this ability, the stem cell may competitively inhibit SARS-CoV-2 from entering and infecting the cell while also promoting anti-inflammatory mechanisms. Future studies should investigate this potent interaction between ACE2 and MSCs or other compatible stem cells that can interfere with ACE2-SP docking of SARS-CoV-2 and modulate immune and inflammatory responses.

Preclinical studies have investigated the use of MSCs in treating lung diseases with similar dysregulation of regular immune and inflammatory responses as COVID-19. In one notable study, the endotoxin model of acute lung injury was used to observe the therapeutic effects of MSC [126]. Mice were treated with MSC 4 h after severe lung damage. MSC treated mice demonstrated significant pulmonary recovery. Significant survival and histological improvements were also observed in MSC-treated groups compared to the control. Additionally, proinflammatory activity was replaced with an anti-inflammatory response to endotoxin after MSC doses were introduced, highlighting the immunomodulatory effects of MSC [126]. Several clinical trials have investigated the therapeutic effects of MSC on lung diseases, including ARDS. Phase 1 dose-escalation study showed no safety issues for the treatment of ARDS with BMSCs (NCT 01775774) [127]. Furthermore, high-dose MSCs improved daily sequential organ failure assessment (SOFA) scores when compared to lower doses. However, when the same group rolled out the phase IIa study, there were no statistical differences between treatment and placebo groups in mortality and the number of ventilator-free days [128]. This discrepancy may have been due to the substantial variation in cell viability observed at the time of injection [128]. Another phase I, double-blind, placebo-controlled trial assessing the safety of human adipose tissue-derived MSC transplantation in patients with ARDS revealed short-term improvement in oxygenation, but ventilator-free days, ICU-free days and duration of hospital stay remained unchanged (NCT01902082) [129].

Although the efficacy of the treatment of lung diseases with MSCs is not yet conclusive, its safety and promising preclinical data warrant further exploration to COVID-19—especially in face of the high morbidity and mortality of the current pandemic. Because of the overlapping pathology of neurological diseases stemming from a harmful inflammatory response and COVID-19-induced pulmonary, cardiovascular, and cerebrovascular disorders, MSCs may provide therapeutic benefits to COVID-19 patients where multiple organ systems are affected.

The envisioned MSC therapy for COVID-19 entails a minimally invasive approach. MSCs injected intravenously (IV) display the propensity to migrate to the lungs making IV administration favorable to treat pulmonary diseases [130, 131]. Another route to consider is intranasal administration. Intranasal administration of MSCs has been tested with neurological diseases, such as stroke, highlighting the homing capability of MSCs [132]. MSCs delivered through the nose have also been shown to be able to restore alveolar growth and vascular development in rat models of bronchopulmonary dysplasia [133]. These relevant findings from other disease indications highlight the many modes of delivery for stem cell therapy for COVID-19, which must be taken into consideration in future clinical trials. Furthermore, because inflammation is an evolving, progressive, and chronic pathology, prolonged treatment through repeated administration of MSCs may be necessary for COVID-19 patients. In this case, less invasive methods like IV injection or intranasal administration are favorable.

The potent immunomodulatory capacity of MSCs renders it a promising option as a novel treatment method for COVID-19. Although there are currently 43 proposed or ongoing clinical trials employing stem cell therapy against Sars-Cov-2 infection, there are no approved MSC-based treatments or therapies. Preliminary results from the US and China demonstrate improved immune function without adverse events in COVID-19 patients treated with MSCs [134, 135]. Intravenous MSC transplantation increases the levels of peripheral lymphocytes and regulatory dendritic cells, with a simultaneous reduction in overactive cytokine-secreting leukocytes, C-reactive protein, and TNF-a [134]. Additionally, delivery of bone marrow-derived MSC exosomes improves patient oxygenation, increases neutrophil and T-lymphocyte counts, and decreases acute phase reactant production [135]. Treatment with human umbilical cord Wharton’s jelly-derived MSCs (hWJCs) similarly improves pulmonary function, normalizes leukocyte counts, and abates acute phase reactant release [136, 137]. MSC-based therapy is also effective in reducing mortality rates among H7N9-induced ARDS patients. The similarities in systemic multi-organ complications between H7N9 and Sars-Cov-2 infections, along with direct evidence of the benefits of MSCs transplantation for COVID-19, further supports the potential of stem cells as an effective treatment [138].

Stem cells are at the forefront of innovative regenerative medicine-based strategies targeting tissue repair and immunomodulation of COVID-19 [139–142]. The use of umbilical cord-derived stem cells [136, 137, 143–146] and bone marrow-derived MSCs [134, 135, 147–150] for targeting neuroinflammation has been documented. The validated safety and efficacy of stem cells in treating respiratory conditions, including ARDS and lung damage, predicts their value for ameliorating COVID-19 outcomes. Furthermore, stem cells effectively repair and regenerate extra-pulmonary tissues damaged by SARS-Cov-2 infection, notably the heart and brain. Perhaps most compelling is that MSCs cannot be infected by SARS-Cov-2, as they are ACE2 negative. Therefore, SP-mediated viral uptake is blocked, allowing healthy MSCs to directly counter inflammation and tissue damage induced by SARS-Cov-2. This ensures that patients treated with MSCs will continue to benefit through the full life cycle of the transplanted stem cells, particularly the sustained anti-inflammatory and regenerative effects. The future of stem cell therapy for COVID-19 is promising, with anticipated positive clinical trial results to come over the next few months.

## The War is Not Over

Since the report of dozens of cases of pneumonia of unknown causes in Wuhan, Hubei Province in December of 2019, COVID-19 has infiltrated across the globe and shaken the lives of the vast majority of the human population. Despite the US leading the world at 2.1 million confirmed cases and 116,130 deaths, many states have begun reopening in response to an economic crisis [1]. Georgia was the first state to begin reopening businesses on April 24th and since then, all 50 states are now in the process of easing restrictions. Amidst this pandemic, another equally devastating threat to the future health of our society is rising: “disillusioned comfort”, or our ignorance of the dangers of the virus that give us a false blanket of security that we may continue to live life as it is. When looking back on the history of pandemics, the 1918 Spanish flu reminds us that human response, or lack thereof, stands as the major determinant for the spread of infectious diseases. Whether the reopening of this country was premature, only time will tell.

Although COVID-19 primary triggers a respiratory disease, pervasive myocarditis and fatal arrhythmia cases in infected patients suggest COVID-19’s deleterious effects on the cardiovascular system. Moreover, a subset of patients also manifests neurologic symptoms likely due to the virus’ ability to retrogradely travel from the lung to the brainstem cardiorespiratory center via neuronal synapses. This multi-organ, heart-brain infection may exacerbate respiratory failure. The critical entry receptor for SARS-CoV-2, the virus responsible for COVID-19, is the ubiquitous ACE2 receptor to which viral SP docks. The ACE2 may reveal acute and chronic multi-organ effects of COVID-19 which warrants further investigation and continued action to lessen the spread of the virus.

As the race for a vaccine continues, anti-viral drugs, convalescent plasma treatment, and stem cell therapies should be explored in tandem to reduce the current mortality rate which ranges from 0.1% to as high as 10% with the elderly (> 65 years of age) and those with pre-existing conditions like hypertension, diabetes, and heart disease [77, 151, 152]. Without access to the vaccine and other proven therapies, the best course of action in the meantime is to continue social distancing, practice good hygiene, and most importantly, check our disillusioned comfort surrounding COVID-19.

## Abbreviations

ACE2: angiotensin-convertingenzyme 2
ANG II: angiotensin II
ARDS: acute respiratory distress syndrome
BASCs: bronchoalveolar stem cells
CDC: Center for Disease Control
CNS: central nervous system
COPD: chronic obstructive pulmonary disease
CT: computed tomography
DPP4: dipeptidyl peptidase 4
ESCs: embryonic stem cells
FDA: U.S. Food and Drug Administration
HEV: hemagglutinating encephalomyelitis virus
hrsACE2: human recombinant soluble ACE2
ICU: intensive care unit
iPSC: induced pluripotent stem cells
IV: intravenous
MERS-CoV: Middle East Respiratory Syndrome-related Coronavirus
MS: multiple sclerosis
MSCs: mesenchymal stem cells
NHC: National Health Commission of China
RBD: receptor-binding domain
RT-PCR: Reverse-transcriptase polymerase chain reaction
SARS-CoV: Severe Acute Respiratory Syndrome Coronavirus
SARS-CoV-2: Severe Acute Respiratory Syndrome Coronavirus 2
SEIR: Susceptible-Exposed-Infectious-Recovered
SP: spike protein
TLR: toll-like receptor
TNF-α: tumor necrosis factor-alpha
UTR: untranslated region
WHO: World Health Organization
